# Pregnancy and associated factors among teenage females in Hula District, Sidama region, Ethiopia: a community-based cross-sectional study

**DOI:** 10.3389/frph.2024.1367436

**Published:** 2024-09-06

**Authors:** Mekdes Amenu, Dansamo Tediso, Tihun Feleke, Selam Fantahun, Endrias Markos Woldesemayat

**Affiliations:** ^1^ Hula Woreda Health Office, Hula Woreda, Eastern Sidama Zone, Sidama Region, Ethiopia; ^2^ Department of public Health, Hawassa College of Health Sciences, Hawassa, Sidama Region, Ethiopia; ^3^ School of Public Health, College of Medicine and Health Science, Hawassa University, Hawassa, Ethiopia

**Keywords:** teenage pregnancy, factors, Hula District, Sidama, Ethiopia

## Abstract

**Background:**

Teenage pregnancy causes serious health, social, and economic consequences, with 95% occurring in developing countries. A significant number of girls start childbearing at an early age in Ethiopia, which contributes to high infant and maternal morbidity and mortality. However, the information on teenage pregnancy and related variables is limited in the study area.

**Objective:**

To assess the prevalence of teenage pregnancy and its associated factors among teenage females in the Hula District, Sidama, Ethiopia.

**Methods:**

A community-based cross-sectional study was employed among 518 teenagers, randomly selected between 15 February and 15 March 2023. An interviewer-administered questionnaire was used for data collection. Bivariate and multivariate logistic regression analyses were applied to assess the relationship under study.

**Results:**

Living in rural areas compared to urban [adjusted odds ratio (AOR) = 3.90; 95% confidence interval (CI): 1.30–11.3], lack of awareness about family planning methods (AOR = 5.90; 95% CI: 1.60–22.24), unfamiliarity with the availability of family planning services (AOR = 3.20; 95% CI: 1.08–9.24), and inadequate communication about sexual issues with parents (AOR = 3.61; 95% CI: 1.14–11.56) were independently associated with teenage pregnancy.

**Conclusion:**

The prevalence of teenage pregnancies in the Hula District was high. Factors such as residing in rural areas, limited access to information on family planning methods and services, as well as a lack of open discussions about sexual health were associated with teenage pregnancy.

## Introduction

Teenage pregnancy is a significant public health concern that poses challenges to maternal and child health (1). Every year, around 16 million adolescent girls between the ages of 15 and 19 give birth, with nearly 95% of these births occurring in underdeveloped nations. These births constitute approximately 11% of the total global births (2). Evidence from a systematic review and meta-analysis indicated that the pooled prevalence of adolescent pregnancy in Africa was 18.8%, rising to 19.3% in the sub-Saharan African region (3). Specific to Ethiopia, the findings showed a teenage pregnancy prevalence ranging from 12.5% to 30.2%. National studies also showed a decline in teenage pregnancy rates in the country, dropping from 16.3% in 2000 to 12.5% in 2016 (4).

Early-age pregnancy poses risks to both the mother and the baby. Young mothers often lack emotional and financial preparedness to care for and raise a child. In addition to social and economic consequences, early motherhood has severe psychological and physical health implications for both the mother and the child. Research has demonstrated that teenage pregnancy is associated, among others, with obstructed labor, pregnancy-related hypertension, fistula, anemia, psychological trauma, low birth weight, and fetal growth retardation leading to high infant and maternal mortality rates (2, 4–9).

Complications arising from pregnancy and childbirth are the leading cause of death among adolescent girls aged 15–19 in impoverished countries. Girls in this age group are twice as likely to die from pregnancy- and childbirth-related complications compared to older women. Furthermore, children born to teenage mothers have a 50% higher likelihood of dying within the first year of life compared to those born to women in their twenties. Alarmingly, only 32% of pregnant teenagers seek antenatal care, and 48% of them seek delivery care, from health professionals (3, 7, 10, 11).

Efforts have been made both globally and nationally to tackle teenage pregnancy. Notably, the World Health Organization (WHO) has issued guidelines aimed at preventing early pregnancy and enhancing reproductive outcomes among adolescents in developing countries, with a particular focus on raising awareness among healthcare providers. Significant progress has been achieved in sexual and reproductive health since the beginning of important initiatives such as the 1994 International Conference on Population and Development (ICPD), Agenda 2063, and the Sustainable Development Goals (SDGs). However, despite these concerted efforts, teenage pregnancy persists as a challenge that requires continued attention and intervention.

Early marriage often leads to an increased risk of teenage pregnancy, and teenage pregnancy can be a consequence of early marriage. Evidence has also shown that poverty, gender inequality, cultural customs and traditions, lack of education and economic opportunities, religion, place of residence, peers and partners' behaviors, family and community attitudes, gender and age, mass media influence, and lack of access to sexual reproductive health (SRH) information and services are contributing factors to the high prevalence of teenage pregnancy (4, 8, 10, 12–14). However, other potentially important factors such as risky sexual behaviors and lack of communication with parents regarding sexual issues have received little attention and have not been adequately addressed in previous studies.

Although several nationwide studies have been conducted, there is a scarcity of studies at the regional level. In the Sidama Region of Ethiopia, particularly in the Hula District, several unique factors make it an important area for studying teenage pregnancy. The district's distinctive cultural norms and practices surrounding marriage and childbearing, combined with its rural nature, significantly influence access to information and reproductive healthcare services. Furthermore, the emphasis on fertility and maternal health service utilization tends to be directed toward the general population of reproductive age rather than teenagers. Moreover, previous studies have been institution-based, lacking adequate representativeness of the general population. Therefore, this community-based study aims to assess the prevalence of teenage pregnancy and associated factors among teenage females in the Hula District.

## Methods

### Study area

The study was conducted in the Hula District, which is one of the 37 districts located in the Sidama Region, situated 96 km south of Hawassa. The Hula District is geographically divided into 2 urban and 17 rural kebeles or wards. According to the data from the 2022 Gregorian calendar, the estimated total population in the district was 103,359, with females accounting for 52,403. Within the female population, approximately 10,246 were teenagers aged 13–19 years. To cater to the healthcare needs of the district, 1 primary hospital, 3 health centers, and 17 health posts are available. In rural areas, health centers and health posts offer primary curative and preventive care, with health workers providing services such as antenatal care, delivery care, and family planning (FP) programs. Similarly, in urban settings, health centers, health posts, and hospitals fulfill the same responsibilities, ensuring access to essential healthcare services. The Hula health office maintains a workforce of 54 health extension workers serving the community.

### Study design, period, and population

A community-based cross-sectional study was conducted in the Hula District from 15 February to 15 March 2023. The source population for this study included all female teenagers aged 13–19 years residing in the Hula District. From this source population, a study population was selected by random sampling of the kebeles in the district. The inclusion criteria consisted of teenagers aged 13–19 years who had been living in the selected kebeles for at least 6 months during the study period. Informed consent was obtained from both the teenage girls and their guardians. However, those who were seriously ill or unwilling to participate were excluded from the study.

### Sample size determination and sampling technique

To determine the sample size, the single population proportion formula was used, with the following assumptions: a 95% confidence level, a 5% margin of error, and an estimated teenage pregnancy proportion of 28.6% (15) based on data in Wogedi District. A 10% non-response rate and a design effect of 1.5 were also considered. As a result, the final sample size was determined to be 518 participants.

The study employed a multistage sampling technique. Initially, all kebeles in the Hula District were stratified into urban and rural areas. Six kebeles were then randomly selected to represent approximately 30% of the teenage population in the district. The estimated number of teenagers aged 13–19 years from these six kebeles was 2,569. The sample size was proportionally allocated to each selected kebele. Health post records were used to create a sampling frame, which included all registered women aged 13–19 years in each selected kebele. The calculated sample sizes of 92, 95, 88, 85, 80, and 78 were allocated proportionally to the respective kebeles: Luda, Gase, Bochessa, Odolakura, Chalbesa, and Hanikomolicha.

To select study participants, a starting point was determined using a lottery method from the sampling frame. Then, simple random sampling techniques were applied within each kebele until the desired sample size was reached. In cases where eligible teenagers were not available in the household, data collectors made repeated visits within the study period, leveraging the labeling and mapping of households that had been done prior to data collection. In addition, when there were multiple eligible teenagers in a household, the interviewee was selected using a lottery method.

### Data collection procedures

Data collection involved face-to-face interviews conducted by six trained nurses (diploma holders) using a structured, interviewer-administered questionnaire and by reading close-ended questions for study participants. The questionnaire was developed based on a comprehensive review of documents, guidelines, and existing literature on similar topics. It covered various aspects such as sociodemographic characteristics, sexual and reproductive characteristics, and individual and peer-level factors. The questionnaire was initially prepared in English, and later translated into the local language, *Sidamu Afoo*, then back-translated to English to ensure consistency. The translations were performed by experts who were competent in both English and *Sidamu Afoo*.

### Data quality control

Data quality control measures were implemented to ensure the accuracy and reliability of the collected data. A properly designed and pre-tested data collection instrument was used. Data collectors and supervisors received training on data collection procedures, the objectives of the study, and the use of data collection tools. A pre-test was conducted in two kebeles, involving 5% of the sample size, to identify any inconsistencies or ambiguities in the questionnaire. Any identified issues were addressed and incorporated into the final version of the questionnaire. Data completeness was checked daily by the supervisor and weekly by the principal investigator to identify and address any missing values.

### Study variables

The primary study outcome was teenage pregnancy, which was determined based on self-reported pregnancy status and confirmed by health professionals (16). Teenage pregnancy was defined as any pregnancy occurring between the ages of 10 and 19 years. The health professionals typically used structured interviews to gather data on self-reported pregnancies to confirm the pregnancies. Hence, pregnancy history was assessed by inquiring whether respondents had ever been pregnant, encompassing pregnancies resulting in live births, stillbirths, or abortions. Adolescents ranged from 10 to 19 years old, encompassing young adolescents (10–14 years old) and older adolescents (15–19 years old). The independent variables included sociodemographic and economic factors (such as age, residence, religion, education level, income level, marital status, parental education, parent occupation, and parent separation), reproductive characteristics (age at first marriage, premarital sex, early age at first sexual encounter, multiple sexual partners, ever had pregnancy, age at first pregnancy, forced marriage, pregnancy planning and desire, knowledge of contraceptives, awareness of where family planning methods are provided, utilization of family planning methods, knowledge of fertile periods for pregnancy, age at menarche, and communication with parents on sexual issues), and individual and peer-level factors (drug/substance use, number of sexual partners, and peer pressure).

### Data processing and analysis

Data processing and analysis involved cleaning, coding, and entry of the collected data into Epi Data version 4.4.1 (Jens, Denmark). The data were then analyzed using SPSS version 25 (IBM, Chicago, IL, USA). Descriptive analysis was performed to summarize the data, and bivariable logistic regression analysis was conducted to identify any statistical associations between each independent variable and the dependent variable. Variables with a *p*-value <0.25 in the bivariable analysis were considered for inclusion in the multivariable logistic regression analysis. Multicollinearity was checked, and the Hosmer–Lemeshow test was used to assess the model's fitness. Adjusted odds ratios (AOR) with 95% confidence intervals (CI) were calculated to determine the presence and strength of associations between predictors and the outcome variable. Statistical significance was considered for *p*-values <0.05.

### Ethical considerations

The study was approved by the Institutional Review Board (IRB) of the Hawassa College of Health Sciences. Ethical clearance was obtained from Hawassa College of Health Sciences under reference number IRB/225/15. Permission to conduct the study was granted in writing by the Hawassa College of Health Sciences and the Hula District health office. The Sidama Eastern Zone Health Bureau Research Ethics Committee approved verbal consent with reference number REC/35/15. The participants were extensively informed about the study's objectives, and verbal consent was obtained from the guardians of teenagers aged 13–17 and from each participant aged 18–19. Stringent measures were taken to ensure the confidentiality and anonymity of all participants, and they were explicitly informed of their right to refuse or terminate the interview at any point. The methods employed adhered to standardized principles, procedures, as well as relevant guidelines and regulations.

## Results

### Sociodemographic characteristics of female teenagers

A total of 518 teenagers participated in this study, with replacement, resulting in a response rate of 100%. The study revealed that the participants' ages ranged from 13 to 19 years, with a mean age of 16.2 (±1.8) and a standard deviation of 1.8. The majority of the respondents (42.10%) were between the ages of 16–17. More than half of the participants (53.30%) resided in urban areas. The majority of the respondents (84.90%) identified as Protestants. In terms of marital status, the majority of the teenagers (84.50%) were single or had never been married. In addition, 68% of the teenagers attended primary school. A significant percentage (78.80%) of the respondents lived with their parents. Regarding income, approximately 45.20% of the participants earned less than 1,000 Birr per month (Table 1).

**Table 1:** Sociodemographic characteristics of teenagers in Hula District, Sidama Region, Ethiopia, 2023 (*N* = 518).

Variables	Categories	Frequency	Percent
Age (years)	13–15	188	36.30
	16–17	218	42.10
	18–19	112	21.60
Residence	Urban	277	53.50
	Rural	241	46.50
Religion	Protestant	440	84.90
	Orthodox	68	13.10
	Muslim	10	1.90
Marital status	Single	438	84.50
	Married	79	15.20
	Widowed	1	0.20
Age at first pregnancy (*n* = 91) (years)	<18	17	18.70
	≥18	74	81.30
Education	No formal education	25	4.80
	Primary school (1–8)	352	68
	Secondary and above	141	27.20
Father's education	Illiterate	155	29.90
	Read and write	203	39.20
	Primary school (1–8)	45	8.70
	Secondary and above	115	22.20
Mother's education	Illiterate	309	59.70
	Read and write	77	14.90
	Primary school (1–8)	55	10.60
	Secondary and above	77	14.90
Father’s occupation	Farmer	353	68.10
	Government employed	102	19.70
	Self employed	60	11.60
	Daily laborer	3	0.60
Mother’s Occupation	Government employed	76	14.70
	Self employed	80	15.40
	Housewife	339	65.40
	Daily laborer	23	4.40
Parental separation/divorce	Yes	110	21.20
	No	408	78.80
Parents’ monthly income (Birr)	<1,000	234	45.20
	1,000–5,000	186	35.90
	>50,000	98	18.90

### Sexual and reproductive health characteristics of female teenagers

**Figure 1:**
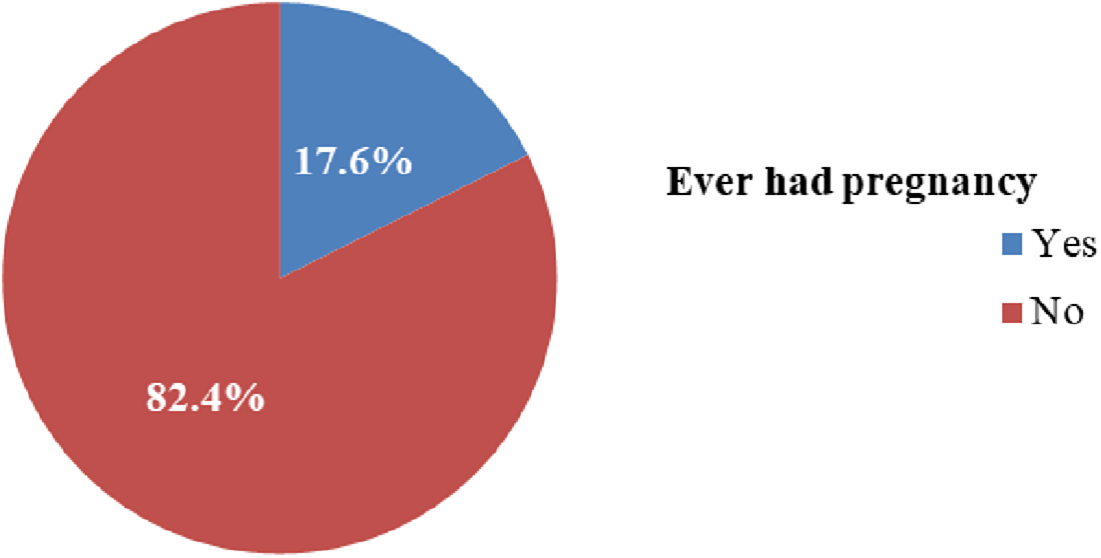
Prevalence of teenagers who were ever pregnant in Hula District.

**Figure 2:**
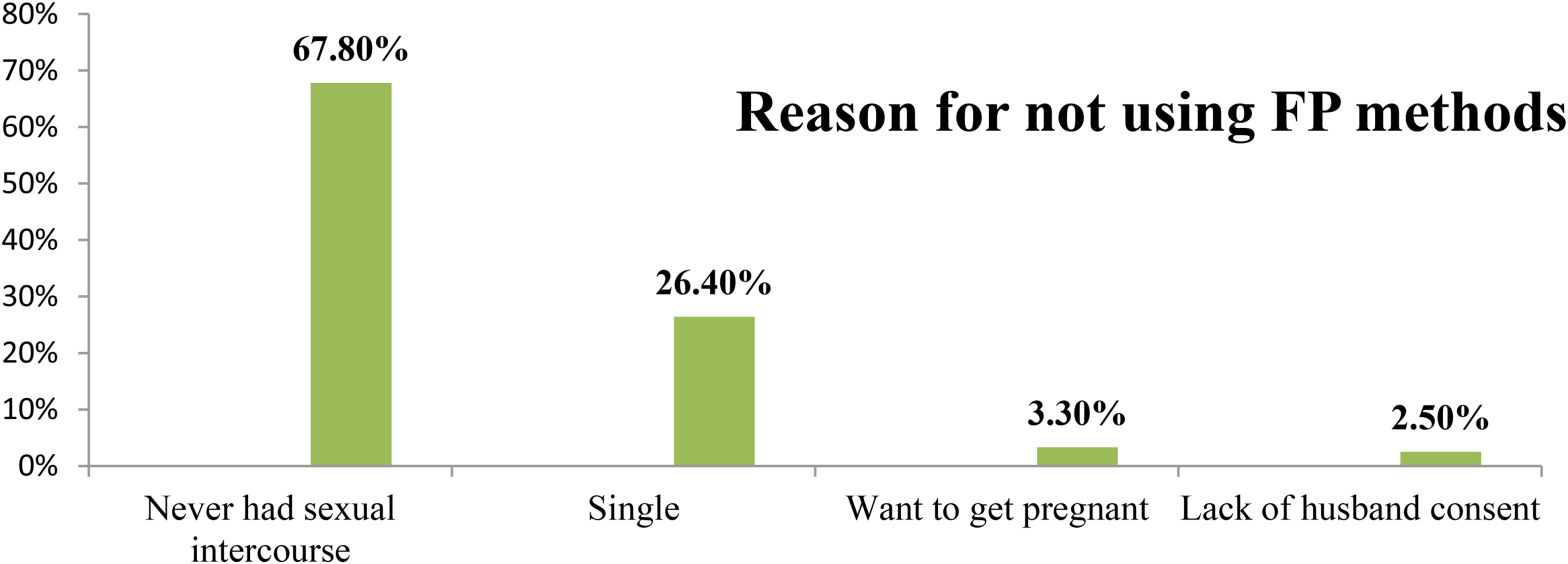
Reason for not using family planning methods in Hula District, Sidama Region, Ethiopia, 2023.

The study results revealed that out of the total respondents, 340 (65.60%) of the individuals had engaged in sexual intercourse, and 91(17.60%) of them had experienced teenage pregnancy (Figure 1). Furthermore, it was found that 131 (38.6%) of the respondents initiated sexual activity before the age of 15 years. In addition, 17 (18.70%) respondents experienced their first pregnancy before age 18 years. Among the majority, 58 (63.70%) respondents had unplanned pregnancies. Approximately half, 253 (48.80%), of the respondents had heard about FP. Among those who were aware of FP, more than half, 143 (56.50%), of the respondents were unaware of any location where FP methods were available. Furthermore, 168 (49.40%) of the respondents had never utilized family planning methods. The primary reason for the non-use of FP methods among 82 (67.80%) teenagers was attributed to never engaging in sexual intercourse (Figure 2). Only 18 (3.50%) of the teenagers were aware of the fertile period during the menstrual cycle. Moreover, over two-thirds, 366 (70.70%), of the teenagers did not communicate with their parents about sexual matters. It was found that more than one-third, 34 individuals (42.50%), of the married teenagers had experienced forced marriages. Lastly, only 60 (11.60%) teenagers reported having married or pregnant peers (Table 2).

**Table 2:** Sexual and reproductive health characteristics of teenagers in Hula District, Sidama Region, Ethiopia, 2023 (*N* = 518).

Variables	Categories	Frequency	Percent
Ever had sex	Yes	340	65.60
	No	178	34.40
Age at 1st sexual intercourse (*n* = 340) (years)	13–14	131	38.60
	15–17	209	61.40
Age at marriage (*n* = 80) (years)	<18	12	15
	≥18	68	85
Multiple sexual partners	Yes	17	3.30
	No	501	96.70
Pregnancy planned and wanted (*n* = 91)	Yes	33	36.30
	No	58	63.70
Ever heard about FP	Yes	253	48.80
	No	265	51.20
Know where FP methods are provided (*n* = 253)	Yes	110	43.50
	No	143	56.50
Ever use FP methods (*n* = 340)	Yes	172	50.60
	No	168	49.40
Know fertile period of the menstrual cycle	Know correctly	18	3.50
	Don’t know	500	96.60
Communication with parents on sexual issues	Yes	152	29.30
	No	366	70.70
Forced marriage (*n* = 80)	Yes	34	42.50
	No	46	57.50
Peers married or pregnant	Yes	60	11.60
	No	458	88.40

FP, family planning.

### Reason for not using family planning methods among teenagers

#### Prevalence of teenage pregnancy

The overall prevalence of teenage pregnancy was 91 (17.60%) (95% CI: 14.00–21).

#### Factors associated with pregnancy of teenage females

Bivariable and multivariable binary logistic regression analyses were conducted to examine the factors associated with teenage pregnancy. In the bivariable logistic regression, several variables such as age, residence, parental separation/divorce, awareness of FP methods, knowledge of where FP methods are provided, prior use of FP methods, and communication with parents on sexual issues were considered as potential factors (with a *p*-value < 0.25) for inclusion in the multivariable logistic regression analysis.

The results of the multivariable binary logistic regression analysis revealed that rural residence, lack of awareness about FP methods, not knowing the location where FP methods are provided, and lack of communication with parents on sexual issues were significantly associated with teenage pregnancy. Accordingly, the odds of experiencing teenage pregnancy among rural residents were 3.90 times higher than among urban residents (AOR = 3.90; 95% CI: 1.30–11.30). Likewise, teenagers who were not aware of FP methods were 5.9 times more likely to experience teenage pregnancy compared to their counterparts (AOR = 5.90, 95% CI: 1.60–22.24). Similarly, teenagers who were not aware of a place to obtain family planning methods were 3.2 times more likely to experience teenage pregnancy compared to those who were aware of a place to obtain family planning methods (AOR = 3.20, 95% CI: 1.08–9.24). Moreover, the odds of experiencing pregnancy among teenagers who did not communicate about sexual issues with their parents were 3.61 times higher than among those who did communicate (AOR = 3.61, 95% CI: 1.14–11.56) (Table 3).

**Table 3:** The COR, AOR, and 95% CI from the bivariate and multivariable logistic regression analysis of factors associated with teenage pregnancy among female teenagers in Hula District, Sidama Region, Ethiopia, 2023 (*N* = 518).

Variables	Teenage pregnancy		COR (95% CI)	AOR (95% CI)
	Yes N	No N		
Age (years)				
13–15	22	166	1.01 (0.48–2.09)	0.93 (0.22–3.90)
16–17	56	162	2.60 (1.37–5.06)	3.10 (0.77–12.37)
18–19	13	99	1	1
Residence				
Urban	30	247	1	1
Rural	61	180	2.80 (1.73–4.49)	**3.90 (1.30–11.30)** *
Parental separation or divorce				
Yes	26	84	1.70 (0.98–2.73)	2.80 (0.72–11.30)
No	65	343	1	1
Ever heard about FP				
Yes	17	236	1	1
No	74	191	5.40 (3.10–9.42)	**5.90 (1.60–22.24)** *
Know place of FP methods provided				
Yes	20	90	1	1
No	71	72	4.4 (2.47–7.96)	**3.20 (1.08–9.24)** *
Ever used FP methods				
Yes	39	133	1	1
No	52	116	1.53 (0.94–2.50)	0.40 (0.11–1.45)
Communication with parents on sexual issues				
Yes	15	137	1	1
No	76	290	2.40 (1.33–4.31)	**3.60 (1.14–11.56)** *

COR, crude odds ratio; 1, reference category.

* Significant at a *p*-value <0.05.

## Discussion

This study aimed to determine the prevalence of teenage pregnancy and its associated factors in Hula District, Sidama Region, 2023, among 518 female teenagers. The findings revealed a high prevalence of teenage pregnancies in the study area. The factors associated with teenage pregnancy included rural residence, lack of awareness about FP, unfamiliarity with the locations where FP methods were provided, and lack of communication with parents about sexual issues.

The overall prevalence of teenage pregnancy was 17.60% in Hula District, Sidama Region, in 2023. These findings were consistent with other studies conducted in South Africa (19.2%) (17) and Latin America (19.1%) (18). This consistency likely arises from similarities in the study populations, as all these studies focused on teenagers.

However, the prevalence of teenage pregnancy in this study was higher than the 13% national prevalence of teenage pregnancy reported in the 2016 Ethiopian Demographic and Health Survey (4), a study conducted in Arba Minch Town (7.7%) (13), Agago District, Uganda (2.8%) (19), and Vietnam (4%) (16). This discrepancy may be due to sociodemographic, cultural, sexual, and reproductive characteristic differences between this study and others, as well as the inclusion of qualitative components in some studies. In addition, differences in outcome measurement could contribute to the disparity. This study relied on self-reporting, which is known to be unreliable and may under report the true burden of teenage pregnancies, whereas in Agago District, Uganda, teenage pregnancy was determined using rapid hCG tests for urine, which may reveal exact results. The teenagers most probably do not report a pregnancy; especially if health professionals gather the information with interviews.

By contrast, the prevalence of teenage pregnancy in the Hula District was lower than in previous studies conducted in Farta woreda, northwest Ethiopia (25.4%) (16), northeast Ethiopia (28.6%) (15), Abia State of Nigeria (49%) (20), and eastern Uganda (35.8%) (21). These differences may be due to the differences in the sociocultural norms and economic factors.

The study found that the odds of experiencing teenage pregnancy among rural residents were higher than among urban residents. This finding aligns with studies conducted in Ethiopia (15, 22) and Tanzania (23). The higher prevalence in rural areas could be attributed to limited access to information and media, along with potential family pressures for early marriage faced by rural women compared to their urban counterparts. In addition, families in rural areas may tend to encourage early marriage, which can contribute to higher rates of teenage pregnancy due to limited education on and restricted access to contraceptives (24).

Teenagers who were not aware of FP methods were more likely to experience teenage pregnancy compared to those who were familiar with them. This finding is supported by studies conducted in Ethiopia (22), California (25), and South Asia (26). Lack of awareness about family planning methods may prevent discussions about the timing of the first pregnancy, and if teenagers were aware of the services, they might delay pregnancy until they desired it (27).

Similarly, teenagers who were not aware of a place to obtain family planning methods were more likely to experience pregnancy compared to those who were aware. This finding is consistent with those from studies conducted in Farta Woreda, northwest Ethiopia (22), and Iran (28). The availability of family planning services plays a crucial role in counseling for intended pregnancies. If teenagers knew where to access these services, it could potentially reduce the occurrence of unplanned pregnancies.

Lastly, the odds of experiencing pregnancy among teenagers who did not communicate about sexual issues with their parents were higher compared to those who did. This finding is in line with previous studies conducted in Deguatembien District, Tigray (29), and Ghana (30). The possible reasons for this include the perception that teenagers are too young for sexual discussions, the taboo nature of the topic, parents' lack of knowledge about what to communicate, and the belief that teenagers already possess sufficient knowledge (31).

### Strength and limitation of the study

The main strength of this study is that it is a community-based study. Another strength is the random sampling that permits to have a representative sample of the study source. However, the study had some limitations. Teenage pregnancy is often underreported by survey participants due to the stigma surrounding early sexual activity and pregnancy during adolescence. This underreporting was partly from the sensitivity and personal nature of the questions asked about sexual behaviors, which can lead to a social desirability bias. In addition, it can be challenging to accurately assess certain health risk factors associated with early teenage pregnancy. Finally, teenagers may either underreport or overreport their exact ages. Therefore, when interpreting the findings, caution should be taken.

## Conclusion

The prevalence of teenage pregnancies in the study area was found to be high. The factors such as living in rural areas, lack of awareness about family planning methods or the locations where family planning services are available, and insufficient communication about sexual matters with parents were significantly associated with teenage pregnancy. Policymakers should consider expanding programs that promote parent–teenage communication regarding reproductive health issues.

Hula District health office, health centers’ management bodies, and health professionals should enhance awareness on FP methods by giving special attention to teenagers in need, with the understanding that it is beneficial to create awareness of the places where family planning methods are provided, offer contraceptive services nearby or make referrals for them, and empower teenage girls, especially rural residents.

Researchers should consider the qualitative part of study to explore other reasons for teenage pregnancy.

## Data Availability

The original contributions presented in the study are included in the article/Supplementary Material, further inquiries can be directed to the corresponding author.
